# NEIGHBORHOOD DISADVANTAGE, FUNCTIONAL DISABILITY, AND COGNITIVE DECLINE AMONG OLDER MEXICAN AMERICANS

**DOI:** 10.1093/geroni/igae098.0597

**Published:** 2024-12-31

**Authors:** Weidi Qin, Lucia Cavanagh, Joshua Owens, Soham Al Snih, Irving Vega

**Affiliations:** University of Wisconsin Madison, Madison, Wisconsin, United States; University of California Los Angeles, Los Angeles, California, United States; University of Florida, Gainesville, Florida, United States; University of Texas Medical Branch in Galveston, Galveston, Texas, United States; Michigan State University Grand Rapids, Grand Rapids, Michigan, United States

## Abstract

This study aims to investigate the longitudinal association between disability and cognitive decline, and to determine the moderating effects of neighborhood disadvantage among older Mexican Americans. Data came from the Hispanic Established Population for the Epidemiologic Study of the Elderly (2004-2005 to 2012-2013) linked to 2010 National Neighborhood Data Archive. Older Mexican Americans without cognitive impairment at baseline were selected (n=1,189). Cognitive function was measured by the Mini-Mental State Examination (MMSE). Neighborhood disadvantage was created based on five census-tract indicators (e.g., % of households below poverty). Disability was indicated by a count of activities of daily living (ADL) and instrumental ADL (IADL) limitations. Multilevel mixed-effects regression models were performed to estimate cognitive change over time, accounting for socio-demographics and health conditions. There was an average decline of 0.91 and 0.32 in the MMSE score as a function of ADL and IADL disability, respectively (p<0.05). Significant interactions between ADL and IADL disability and neighborhood disadvantage were observed. Among older Mexican Americans who were exposed to higher levels of neighborhood disadvantage, ADL and IADL disability were significantly associated with faster cognitive decline. The study findings suggest that neighborhood disadvantage undermines cognitive health among older Mexican Americans who have functional disability.

